# Use of the supportive and palliative care indicators tool (SPICT™) for end-of-life discussions: a scoping review

**DOI:** 10.1186/s12904-024-01445-z

**Published:** 2024-05-16

**Authors:** Melanie Mahura, Brigitte Karle, Louise Sayers, Felicity Dick-Smith, Rosalind Elliott

**Affiliations:** 1 Avondale University, Wahroonga, Sydney, NSW Australia; 2 Hammond Care, Greenwich, Sydney, NSW Australia; 3 Royal North Shore Hospital, St. Leonards, Sydney, NSW Australia; 4 University of Technology Sydney, Ultimo, Sydney, NSW Australia

**Keywords:** Communication, Documentation, Palliative Care, Patient Care Planning, Terminal Care

## Abstract

**Background:**

In order to mitigate the distress associated with life limiting conditions it is essential for all health professionals not just palliative care specialists to identify people with deteriorating health and unmet palliative care needs and to plan care. The SPICT™ tool was designed to assist with this.

**Aim:**

The aim was to examine the impact of the SPICT™ on advance care planning conversations and the extent of its use in advance care planning for adults with chronic life-limiting illness.

**Methods:**

In this scoping review records published between 2010 and 2024 reporting the use of the SPICT™, were included unless the study aim was to evaluate the tool for prognostication purposes. Databases searched were EBSCO Medline, PubMed, EBSCO CINAHL, APA Psych Info, ProQuest One Theses and Dissertations Global.

**Results:**

From the search results 26 records were reviewed, including two systematic review, two theses and 22 primary research studies. Much of the research was derived from primary care settings. There was evidence that the SPICT™ assists conversations about advance care planning specifically discussion and documentation of advance care directives, resuscitation plans and preferred place of death. The SPICT™ is available in at least eight languages (many versions have been validated) and used in many countries.

**Conclusions:**

Use of the SPICT™ appears to assist advance care planning. It has yet to be widely used in acute care settings and has had limited use in countries beyond Europe. There is a need for further research to validate the tool in different languages.

**Supplementary Information:**

The online version contains supplementary material available at 10.1186/s12904-024-01445-z.

## Key message

What is already known on this topic?

• The SPICT^™^ was developed to assist clinicians to screen patients for palliative care needs.

What this review found.

• The SPICT™ assists conversations about advance care planning and facilitates changes in documented goals of care.

• The SPICT™ is available in at least eight languages (and used in many countries.

How the findings of this review may affect practice and research.

• Evidence suggests that formalising screening for palliative care needs using the SPICT™ is advantageous for advance care planning; clinicians should consider using the SPICT™ to initiate discussions with people with life limiting conditions.

• Further research is required to validate the tool in different languages and extend its use in acute care settings and with other patient cohorts.

**Supplementary Information:**

The online version contains supplementary material available at 10.1186/s12904-024-01445-z.

## Introduction

The demand for palliative care services globally has outpaced service availability, particularly in low and middle-income countries [1]. This is expected to continue as the population ages and the burden of noncommunicable disease increases. Thus, non-specialist palliative care health professionals may be required to manage care. The Supportive And Palliative Care Indicators Tool (SPICT™) [2] is one instrument available for non-specialist palliative care clinicians which may assist them in assessing unmet palliative needs and care planning.

Evidence suggests that clinicians feel inadequately prepared to conduct end-of-life discussions with patients who are terminally ill [3–5] and are also unsure of the appropriate time to start these discussions or whether to involve a specialist palliative care team [5–7]. Clinicians have reported their discomfort when addressing the topic of death with seriously ill patients [5].

From the perspective of patients with advanced illness, honest information from a trusted health care professional is the preference of most [7]. A survey study conducted in Canada involving 434 patients with advanced illness found over half of patients felt it was ‘very important’ to have a sense of control over decision-making regarding their care and 56% felt it was ‘extremely important’ not to be kept alive on life support if there was little hope of recovery [7]. The default medical decision to do everything to save life may be contributing to delays in referral to a specialist palliative care team, burdensome medical treatment and poorer quality of life for many patients [8]. Thus, a standardised, reliable and validated method of assessing and planning care in collaboration with the patient is required.

The terms ‘end-of-life’ and ‘terminally ill’ have been conceptualised as synonymous and ‘apply to patients with progressive disease with months or less of expected survival.’ [9]. In the United States there is consensus that referral to specialist palliative care services is required at the time of diagnosis for patients with neurologic disease, frailty, multimorbidity, advanced cancer, organ or cognitive impairment, patients with a high symptom burden and patients with onerous family or caregiver needs [10]. However with an ageing population and increased levels of dementia and frailty non-palliative care clinicians need a tool with a common language to identify those who are nearing the end of life and to promote a palliative approach to care. According to the High Authority for Health, an independent organisation that promotes quality outcomes in the fields of health, sociology and medicine a palliative approach is, “a way of addressing end-of-life issues early on: make time to talk about ethical questions, psychological support, comfort care, the right care, and give a timely thought to the likely palliative care needs of people approaching the end-of-life.” [11], p1.

Advance care planning, “a process that supports adults at any age or stage of health in understanding and sharing their personal values, life goals, and preferences regarding future medical care” [12] is one aspect of palliative care often provided by medical professionals which may assist in ensuring people’s needs are met, and care and communication are enhanced. Early advance care planning is vital, particularly for patients with neurodegenerative conditions before they lose capacity to express their wishes [8] “to help ensure that people receive medical care that is consistent with their values, goals and preferences during serious and chronic illness.” [12] Research has revealed that patients who have had the opportunity to discuss their preferences at the end-of-life are more likely to receive care that is consistent with those preferences. Findings also include greater patient and carer satisfaction and less conflict regarding decision making when end-of-life preferences have been examined [13].

People who have life limiting conditions may benefit from the delivery of advance care planning using a systematic approach. The SPICT™, although not designed for this purpose may enhance the approach particularly when health professionals who have limited palliative care experience are required to facilitate advance care planning.

The SPICT™ [2] was designed to identify patients at risk of deteriorating or dying and to screen for unmet palliative care needs. The tool includes general indicators of deterioration and clinical indicators of life-limiting conditions. The accompanying SPICT™ guide provides prompts and tips and a suggested framework (REMAP Ready, Expect, Diagnosis, Matters, Actions and Plan) [14] for conducting future care planning conversations. The tool is reported to be simple to use and designed for use by all multidisciplinary team members in any care setting [13].

The SPICT™ was evaluated using a mixed methods participatory approach [2]. Peer review and consensus was gathered for the 15 revisions of the SPICT™ over an 18-month period. Each iteration of the tool was distributed to clinicians and policy makers internationally until consensus was reached [2]. The research team worked concurrently with clinicians in four participating units at an acute tertiary hospital in Scotland to screen all patients with advanced organ disease whose admission to hospital was unplanned (*n* = 130) using a checklist that included the SPICT™ general indicators, disease specific indicators and the surprise question (SQ), “Would you be surprised if this patient were to die in the next 6 to 12 months?”. Data were gathered over an 8-week period and patients were followed up for 12 months [2]. A significantly greater number of patients who died at 12-months had two or more admissions in the previous 6 months before being screened. These patients also had increased care needs and persistent symptoms despite optimal treatment. The researchers proposed that better identification, assessment and pre-emptive care planning could reduce the risk of unplanned hospital admission and prolonged inpatient stays [2]. Of note the patients’ diagnoses were limited to advanced illness which was non-malignant and ethnicity was homogenous [2]. The SQ was removed from subsequent versions of the SPICT™ and the rationale for removing it remains unclear. The SPICT™ continues to be revised and versions are available in different languages [2].

The intention of this review was to examine the impact of the SPICT™ on advance care planning and the extent of its use. The patient cohorts, languages, and contexts in which the SPICT™ is available and used were examined.

### Review questions

The following primary question was addressed:

1. How does use of the SPICT™ assist with conversations about advance care planning?

Secondary review questions were:

2. What is the extent of the use of the SPICT™ (which patient cohorts, contexts, and countries is it used)?
3. In which languages has the SPICT™ been validated?
4. Does use of the SPICT™ facilitate changes in documented goals of care?

### Design and methods

This scoping review was performed in accordance with the Joanna Briggs Institute Manual for Evidence Synthesis Scoping Review Framework [15] and the Meta-Analyses Scoping Review extension for scoping reviews (PRISMA-ScR) checklist [16] was used to guide the reporting.

### Preliminary literature search

An initial search focussed on inpatients with a chronic illness nearing the end of life however the search was expanded to include all care settings where the SPICT™ was being used for adults with a life-limiting chronic illness to evaluate its efficacy in advance care planning. Thus the search reflected the International Association for Hospice and Palliative Care definition of palliative care “the active holistic care of individuals across all ages with serious health-related suffering due to severe illness, and especially of those near the end of life.” [17]. A life-limiting illness or condition encompasses both malignant and non-malignant diseases as well as the effects of ageing.

A preliminary search of EBSCO Medline, the Cochrane database of systematic reviews, Prospero and JBI Evidence Synthesis was conducted in June 2022. No current or planned systematic or scoping reviews specifically on this topic were identified. A systematic review by Teike Luthi, et al. [18], examining instruments for the identification of patients in need of palliative care in the hospital setting was identified. The current scoping review differs from the systematic review by Teike Luthi, et al. [18] as the aim was to identify and describe all research related to how the SPICT™ is used in end-of-life discussions and what influence this has on advance care planning and goals of care.

### Inclusion criteria

#### Participants

The population of interest was adult patients (> 18 years) with a life-limiting chronic illness.

#### Concept

The concept of interest was the SPICT™. Any studies incorporating the SPICT™ were included in this review since its development in 2010. Studies evaluating the SPICT™ for prognostication purposes were excluded as this was not the intent of this review.

#### Context

Published and unpublished studies in any language for which a translation could be obtained were included. Published and unpublished studies in any setting that met the eligibility criteria were included.

### Evidence sources

This scoping review included both experimental and quasi-experimental study designs. In addition, analytical observational studies including prospective and retrospective cohort studies, case-control studies and analytical cross-sectional studies were considered for inclusion. Systematic reviews that met the inclusion criteria were included. Qualitative studies, theses and dissertations were also considered if they met the inclusion criteria.

### Search strategy

An initial search on this topic in the EBSCO Medline and PubMed databases was reviewed for relevant abstracts and titles to determine keywords and index terms. MESH terms used in the final search strategy included: Communication; Documentation; Palliative Care; Patient Care Planning; Advance Care Plan; Decision Making and Chronic Disease. The research abstract for this scoping review was registered on the Center for Open Science website (10.17605/OSF.IO/DN27C) in August 2022 prior to performing the definitive search in September. The search was conducted on 28th September 2022 and date limited i.e., 2010-September 2022. The database and grey literature searches were updated on 27th January 2024 to identify further studies published beyond this date.

Electronic databases searched included EBSCO Medline, PubMed, EBSCO CINAHL, APA Psych Info, ProQuest One Theses and Dissertations Global. Publications listed on the SPICT website (www.spict.org.uk) were cross checked with the records included from the electronic databases, duplicates were removed and further records were added to the Endnote library for screening. Reference lists of included studies were reviewed for additional studies.

All websites searched for additional records (grey literature sources) are included in supplementary file 1. The expanded search strategy for the EBSCO Medline database is also provided in supplementary file 1.

### Study selection

All records were collated in an EndNote library. Duplicate records were removed manually by RE. The screening process involved two independent reviewers (MM and RE) reading titles and abstracts. Full text screening was conducted independently by the same two reviewers. Any discrepancies between the two reviewers at each stage of the process was resolved following review and consultation of a third reviewer (BK). Studies that did not meet the inclusion criteria were excluded with a reason recorded. Data extracted from included studies has been recorded in the standardised data extraction form (supplementary file 2). Critical appraisal of included studies was not performed and thus studies were not excluded based on methodological quality.

### Data synthesis

Key aspects of the included studies were summarised in tables. Also consistent with the approach for a scoping review a textual narrative synthesis [19] was performed with the primary aim of addressing the review questions.

## Results

**Fig. 1:**
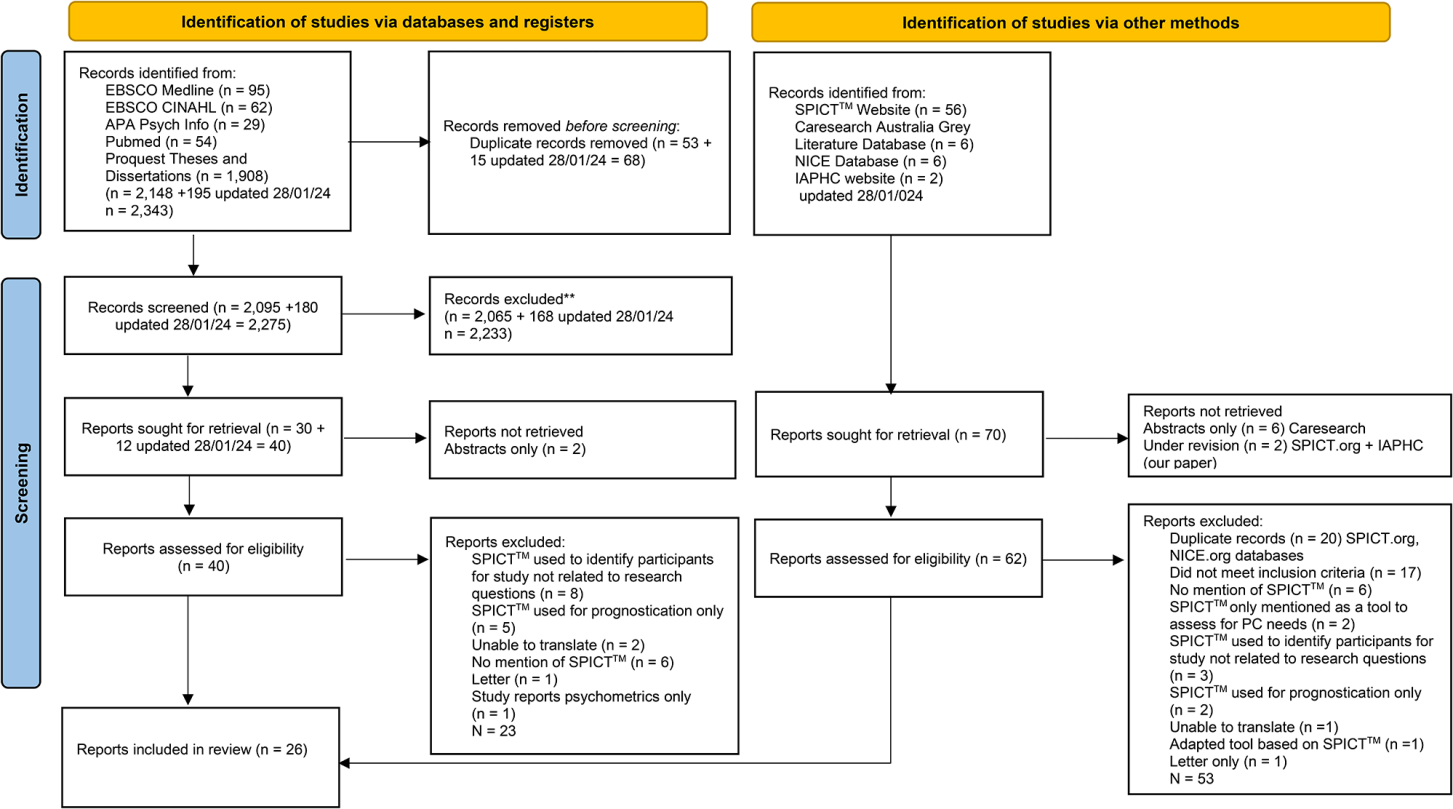
Flow diagram of number of records retrieved, screened, excluded and included **Abstract and title screening involved assessing each record for relevance to the review questions i.e., if no mention of the SPICT™ or/and advance planning conversation the record was excluded from further consideration

Over 2,000 records were retrieved. Five guidelines and six conference abstracts were found but these either did not relate to the review questions or did not contain sufficient information to be included. After applying the exclusion criteria 26 reports were included in this scoping review. The flow diagram (Fig. 1) presents the number of records retrieved, screened, excluded and included.

There were multiple study designs including validation and translation (*n* = 8) studies [20–27] and clinical improvement projects (*n* = 3) [28–30]. The focus of the clinical improvement projects was to increase the identification of palliative care needs and care planning through the use of the SPICT™. Two reviews (one of these included a survey study) ^18,31^ and two theses were included ^28,29^ (Table 1).

**Table 1:** Characteristics of included studies and reviews

**Author**	Study Type	Population	**Concept**	Context	Outcome related to research questions:			
					1	2	3	4
Afshar, et al. ^27^	Prospective exploratory feasibility study	10 GPs in rural and urban settings with no experience of SPICT-DE™ 50% male Mean age 46 years 79 patients > 18 years with a life-limiting disease 50.6% female Mean age 79 years	SPICT-DE™ (German)	General practice Germany	x	x	x	x
Afshar, et al. ^32^	Translation with TRAPD Model + Mixed methods study with pre-post design	5 GPs for pre-post test Focus groups: Group A = 15 Group B = 13 (all GPs)	SPICT-DE™ 2017 (German)	General practice Germany		x	x	
Bergenholtz, et al. ^18^	Translation with TRAPD model, + Qualitative analysis of survey and focus groups	12 nurses 12 health care workers 5 doctors with experience caring for patients with life-limiting disease	SPICT-DK™ (Danish)	General practice, primary care, one hospital Denmark		x	x	
Casale, et al. ^19^	Translation with Beaton (2000) protocol and WHO guidelines + content validity	71 GPs completed questionnaire to identify patients who were SPICT™ positive (+) 73,526 patients 1,303 patients > 18 years (1.7%) were SPICT +	SPICT-IT™ (Italian)	General practice Italy		x	x	
Clark ^23^	Practice Improvement Project	2 x Nurse Practitioners 50 patients with heart failure, COPD or renal disease	SPICT™ 2018	Primary health care regional clinic USA	x	x		
Chan, et al. ^30^	Retrospective audit of electronic medical records	227 patients with advanced cancer 59% female, 63% black Mean age 66 years	SPICT™	Acute care hospital USA		x		
Dinega ^24^	Clinical improvement project	48 patients SPICT™ positive during project 18 and 23 registered nurses respectively on unit and float pool 13 hospital physicians on unit Gender and age of participants not reported	SPICT™ 2015	Long term care cardiopulmonary unit USA	x	x		x
Effendy, et al. ^35^	Descriptive cross-sectional survey of patients with non-communicable disease (NCD)	124 adult patients with NCD 76 patients were SPICT™ + (61%) 51% female Mean age 53 years	SPICT-In™ (Indonesian)	Acute care hospital Indonesia		x	x	
Fachado, et al. ^17^	Translation with Beaton (2000) protocol + validation of psychometric properties	30 health professionals 188 patients with a life limiting disease Mean age 83 years	SPICT-ES™ (Spanish)	Primary care, palliative care and residential aged care Spain		x	x	
Fumaneeshoat, et al. ^33^	Retrospective cross-sectional study of patients presenting to outpatient clinics	9,990 participants over 14 years 781 patients were SPICT™ + (7.8%) 36.3% male Mean age 59 years	SPICT™ Low Income Settings (LIS)	Outpatient clinics (medical, surgical & radiation oncology) Thailand		x	x	
Hamano, et al. ^20^	Translation using the Beaton (2000) protocol + single centre cross-sectional observation study	87 patients > 65 years 8 patients SPICT™ + (9.2%) Mean age 79 years	SPICT-J™ (Japanese)	Rural ambulatory clinic Japan		x	x	
Krause, et al. ^37^	Delphi study to develop the SPICT-SA™	15 members of expert advisory group 43 health care professionals surveyed Draft formulated by expert advisory group and re-distributed 14 surveys received back, no further changes	SPICT-SA™ (South African)	South Africa		x		
Liyanage, et al. ^29^	Prospective feasibility study mixed-methods design	2 Directors of Nursing with minimum of 12 months experience in aged care facility 187 residents of high and low care at two facilities 83% male Mean age 82 years	SPICT™ 2012	Residential aged care Australia	x	x		x
Lunardi, et al. ^28^	Prospective cohort study	31 renal nurses 100% female Mean age not reported 152 patients of these 25 (16%) were SPICT™ positive Mean age/gender not reported	SPICT™ 2019	Specialist renal ward Australia	x	x		
Maas, et al. ^26^	Mixed methods study involving a systematic literature review and survey of European practice	One GP member of the European Association for Palliative Care Taskforce in Primary Care for each of the nominated 14 countries	SPICT™	General Practice 14 European countries		x		
Pairojkul, et al. ^36^	Point prevalence survey of patients’ medical records	5,763 patients surveyed 1,079 SPICT + (18.7%) 54% male Mean age 63 years	SPICT ™2017	14 tertiary care government hospitals Thailand		x		
Pham, et al. ^21^	Translation using the Beaton (2000) protocol + Qualitative analysis of focus groups	2 Physicians 2 GPs 13 registered nurses	SPICT-SE™ (Swedish)	Primary care and one university hospital Sweden			x	
Pinedo-Torres, et al. ^38^	Analytical cross-sectional study	172 adult patients with chronic disease 123 patients were SPICT™ + (71.5%) 54.7% male Mean age 61 years	SPICT-ES™ (Spanish)	Acute care hospital Peru		x	x	
Sripaew, et al. ^22^	WHO guidelines for translation and cross-cultural adaptation	1st iteration: 7 GPs 11 primary care nurses 3 pharmacists 7 other health care workers (e.g., nursing assistants) 87% female 2nd iteration: 11 GPs 23 palliative care nurses 71% female	SPICT™ Low Income Settings (LIS) Thai version	Community health providers and multidisciplinary palliative care providers Thailand			x	
Sudhakaran, et al. ^34^	Cross-sectional study of households in the community	2,041 participants 88 identified as needing palliative care (4.31%) 51.2% female Mean age 61 years	SPICT-4ALL™	Community dwellings India		x		
Teike Luthi, et al. ^16^	Systematic review of measurement properties of instruments used for adult patients in hospital to assess general or specialist palliative care need	N/A	SPICT™	Review – multiple countries Authors based in Switzerland			x	
van Baal, et al. ^25^	Prospective interventional mixed-methods study with pre-post design (Phase 3 of the OPAL study)	45 GPs 71% male Pre-intervention = 302 deceased patients 48% female Mean age 82 years Post-intervention = 154 deceased patients (> 18 years with chronic progressive disease) 54.5% female Mean age 84 years	SPICT-DE™ (German)	General practice Germany				x
van Wijmen, et al. ^31^	Prospective cohort study of two general practices in the Netherlands	GP practice A – 2,160 patients GP practice B – 1,480 patients	SPICT-NL™ (Dutch)	General Practice Netherlands		x		

Notes: GP = general practitioner; SPICT = supportive and palliative care indicators tool, WHO = World Health Organisation

### How does use of the SPICT™ assist with conversations about advance care planning?

Research reveals that the SPICT™ appears to assist clinicians with conversations about advance care planning by providing a proforma for essential aspects of end-of-life care, a framework for end-of-life conversations and a common language to collaborate within the multidisciplinary team.

For example, in a prospective exploratory feasibility study to explore the practical use of the SPICT™ resulted in increased palliative care planning [32]. In this study general practitioners (GPs) [*n* = 10] were trained in the use of the German version of the SPICT™ (SPICT-DE™) and during a two-month intervention period were asked to use the tool with any adult patients diagnosed with a life-limiting disease (*n* = 79) and these patients were followed up at 6 months. The GPs’ actions as recommended by the SPICT-DE™ were considered appropriate with the most frequent actions being “Agree a current and future care plan with the person and their family; support family carers” (*n* = 59 [75%)),“Review current treatment and medication to ensure the person receives optimal care; minimise polypharmacy”(*n* = 53 [67%]), and “Plan ahead early if loss of decision-making capacity is likely”(n = 49 [62%]). Of note “Consider referral for specialist palliative care consultation to manage complex symptoms” was considered appropriate for 25 (32%) patients. The effect of the SPICT™ was evident at the 6-month follow-up; the most frequently initiated actions were “Review current treatment and medication to ensure the person receives optimal care; minimise polypharmacy” (n = 36 [46%]) and “Plan ahead early if loss of decision-making capacity is likely” (n = 29 [37%]).

Further implementation research by Afshar et al. [33] with GPs in Germany revealed that GPs considered that the tool supported the communication and coordination of care and considered it broadened their perspectives of the meeting the needs of people especially those with non-cancer diagnoses. Of note over 50% of patients in this study had their agreed care plan initiated at the 6-month follow-up. Some GPs who had extensive experience and training claimed that the tool had no effect on their practice. However overall more than two thirds of the sample reported that they could envisage using the SPICT-DE™ in everyday practice.

In addition, three studies found that nurses who were trained to use the SPICT™ increased their self-efficacy in identifying patients who may be nearing the end of life and promoted an advance care plan discussion with these patients ^28 29 34^.^21−23^ In the study set in a renal ward, patients were screened on admission to identify those nearing the end of life by nurses using the SPICT™ [34]. An alert was added to the ward patient name list when a patient was identified as nearing the end of life (‘SPICT™ positive’) which prompted a review by the physician and multidisciplinary team. In this study 16% (25/152) of newly admitted patients were screened as ‘SPICT™ positive’; all of these patients received a palliative care consult and were discharged with an advance care directive including a resuscitation plan [34]. Incidentally nurses reported a significant increase in their ability to identify patients approaching end of life.

Similarly high SPICT™ screening rates and end of life conversations and referrals were revealed in a clinical improvement project designed to improve palliative care screening and consultation on admission to the cardiopulmonary unit of a long-term acute care facility using the SPICT™ [29]. In this project involving patients requiring mechanical ventilation and cardiac monitoring, 83% (59/71) of nurses working in the unit were trained in the use of the tool and screened all 50 newly admitted patients in the study period, 48 of whom were ‘SPICT™ positive’. Only 7 received a palliative care consultation within a week of admission however all 7 of these patients received a resuscitation plan and an advanced directive. Of note the use of the SPICT™ for screening resulted in a doubling of the facility’s monthly average number of palliative care referrals (from 32 to 84). In another clinical improvement project designed to increase screening and referral for palliative care among ambulatory care patients, nurse practitioners found the SPICT™ ‘.*opens the door to a discussion of palliation*.’ and was ‘.*helpful in determining eligibility for palliative care.*.’ p 22 ^28^. This project using both quantitative and qualitative approaches revealed an increase in palliative care referrals from 16% (*n* = 8/50) to 50% (*n* = 25/50) after the SPICT™ was introduced.

Two studies designed to translate and validate the SPICT-DK™ (Danish) [21] and SPICT-SE™ (Swedish) [24] involving focus groups with health care professionals revealed positive responses from doctors and nurses. The tool was described as a linguistic framework among these professionals and that use of the SPICT™ gave them a common language in which to collaborate and focus when treating and caring for patients [21]. The specificity of the tool was highlighted by nurses and medical doctors [24].

Conversely the expert committee comprising family physicians and palliative and home care specialists who provided input to the translation and cross-cultural adaptation of the SPICT™ into Japanese were more circumspect [27]. These experts were concerned that the tool might not be appropriate for framing advance planning conversations as a ‘not-telling the truth’ culture was prevalent and health care was heavily siloed into specialities so that care planning was fragmented.

### What is the extent of the use of the SPICT™ (which patient cohorts, contexts, and countries is it used)?

The SPICT™ has been used to screen for palliative care needs in many patient cohorts, settings and countries. The cohorts in which the SPICT™ has been used include people over 65 years [35], those with advanced cancer ^32,36^ and with chronic diseases including cardiovascular disease [28], renal disease [34] and pulmonary disease [29].

Ten of the included studies were conducted in primary care and general practice settings ^20–22,24,25,30–32,37,38^. The SPICT™ was also used in outpatient clinic settings ^23,28,39^ and residential aged care ^29,35^. One cross sectional survey of community households in India used the SPICT™ to identify patients with palliative care needs in two rural communities [40]. The SPICT™ was originally developed for use in a hospital setting but not formally validated during its development [2]. All of the contexts in which the SPICT™ has been used are listed in Table 1.

Of the included records ten were studies conducted in European countries ^20–22,24,30–33,37,38^; seven in Asia ^23,25,27,39–42^; three in the USA ^28,29,36^; two in Australia ^34,35^; one in South Africa [43] one in Chile [26] and one in Peru [44], and one paper was a review performed by authors based in Switzerland [18]. Of note the systematic review and survey of European primary care GP practice to identify patients for palliative care revealed that the United Kingdom was the only European country at the time that incorporated the SPICT™ to identify palliative care needs in primary and secondary care in clinical guidelines [31].

### In which languages has the SPICT™ been validated?

The SPICT^™^ has been translated, cross culturally adapted and validated to identify patients with palliative care needs in Danish [21] and German [38] using the Translation, Review, Adjudication, Pre-testing and Documentation (TRAPD) model. Another study by Afshar, et al. [32] further established the validity of the SPICT-DE^™^ in German in general practice with a patient cohort. In addition the SPICT^™^ has been translated from English to Italian [SPICT-IT^™^] [22], Spanish [SPICT-ES^™^] [20], Swedish [24] and Japanese [SPICT-J^™^] [23] using the Beaton protocol for cross cultural adaptation of health measures [45]. Farfán-Zuñiga and Zimmerman-Vildoso [26] established the reliability and validity of the SPICT-ES^CHTM^ after culturally adapting the SPICT-ES^™^ using the Beaton protocol. Nurses positively evaluated the feasibility of the tool. In addition Oishi et al. [27] also performed a translation and cross-cultural adaptation of the SPICT^™^ into Japanese using a similar approach. The forward-back translated Indonesian version of the tool was found to be highly reliable and valid and greatly assisted in identifying hospital patients’ unmet palliative care needs [41].

The SPICT™ for low-income settings (LIS) was translated and cross culturally adapted for use in Thailand [25]. The interrater reliability of the final SPICT-LIS™ Thai version was high when nurses and GPs used it to ascertain palliative care needs of patients in case vignettes.

A Delphi study was used to develop the SPICT™ for the South African context [43]. Modifications to the original tool included the addition of haematological and infectious diseases and trauma however the SPICT-SA™ has yet to be validated in these patient cohorts. Although not a validated study per se in research comparing the performance of the Dutch version of the tool (SPICT-NL™) and the SQ in general practice (*n* = 3,640) the SPICT™ was found to be superior to the SQ in identifying patients with palliative care needs particularly younger people [37].

Of note the SPICT4-ALL™ [46] is a simplified version of the original SPICT™ developed for family/friends and care staff to identify individual palliative care needs. It is available to download from the SPICT™ website in English, German, Danish and Spanish. Although Sudhakaran, et al. [40] successfully used it to identify palliative care needs in two communities in rural India. No studies validating or evaluating it were found in our search.

### Does use of the SPICT^™^ facilitate changes in documented goals of care?

There is evidence that the SPICT™ by virtue of assisting clinicians to discuss end of life care facilitates changes in documented goals of care. Specifically this was demonstrated in a pre-post intervention study in which GPs trained in palliative care and the use of the SPICT-DE™ were requested to use it in their everyday practice for 12 months with every adult patient diagnosed with a chronic, progressive disease [30]. This occurred concurrently with a public campaign focused on informing health care providers and stake holders in two counties in Germany about end-of-life care. GPs’ documentation improved after the intervention. Records of care planning increased from 33 to 51% and documentation of preferred place of death towards the end-of-life increased from 20 to 33% and patients’ wishes, and spiritual beliefs increased from 18 to 27%. Incidentally GPs’ self-reported quality of end-of-life care increased after the implementation of the SPICT-DE™ and the information campaign [30].

In a study including 187 residents in an aged care facility in Australia comparing the SPICT™ and SQ, two Directors of Nursing pre-screened residents using the SQ and if the response was ‘yes’ (SQ+) applied the SPICT™ [35]. Of the 80 (43%) residents who were SQ+, 100% of these showed signs of nearing end of life according to the SPICT™. Of these residents 39 (49%) had some form of palliative care from either GPs, a specialist palliative care physician or palliative care nurse. Nearly all 39 (97%) had a GP management plan, and 67% had an advance care directive and 67% had discussed treatment options with their care provider [29]. It is unclear whether the SPICT™ affected care planning or documentation as the study involved pre-screening with the SQ and documentation was not assessed before and after this intervention.

## Discussion

Death and dying are taboo in many countries and thus any discussion about end of life is challenging. However, clinicians are morally and ethically obliged to appropriately initiate discussions about advance care planning towards the end of life when patients are ready [47]. This review found that the SPICT™ may help the clinician with this conversation. Specifically, evidence suggests that the tool may be a useful proforma and a conversation ‘checklist’ to ensure that the priority areas for advance care planning are addressed. Specifically, the tool may enable an assessment of the person’s readiness to have an advance planning conversation and an exploration of their expectations, the diagnosis, what matters to them, treatment options and future plans [14]. Importantly extensive specialist training is not required to administer it; the studies in this review employed brief information interventions to prepare clinicians to use the SPICT™. Thus, the SPICT™ provides a method of ‘objectively’ assessing palliative care needs, articulating the requirement for a specialist palliative review if required and advance care planning.

This review found that the SPICT™ was used in mainly primary health care settings and predominately in European countries. Of note there were few published records of its use in countries in the Asian and African continents and North America. The tool has been translated into more than eight languages including Spanish (SPICT-ES™) [20], Italian (SPICT-IT™) [22], German (SPICT-DE™) [38] and Japanese (SPICT-J™) [23] although not all versions have been formally validated [25–27, 33]. 

Furthermore, there is evidence to suggest that the SPICT™ may facilitate changes in the goals of care and documentation of end of life care planning and patient wishes. Incidentally the SPICT™ appears to be positively received by clinicians with some suggesting that the tool provides a common language for clinicians when collaborating to identify palliative care needs and provide palliative care.

Of note the tool did not meet the COnsensus-based Standards for the selection of health Measurement INstruments (COSMIN) criteria [18]. However arguably these criteria may not be the most appropriate criteria on which to base an assessment of the SPICT™ given that it was never meant to be used to objectively measure a parameter such as prognosis; Highet et al. [2] were clear about the remit of the tool i.e., “help clinicians working in primary and secondary care recognise when their patients might be at risk of dying and likely to benefit from supportive and palliative care in parallel with appropriate ongoing management of their advanced conditions.” [2], p11.

There is an imperative to improve recognition of palliative care needs particularly in fast paced acute care settings. Evidence suggests that a tool such as the SPICT™ is an important adjunct for initiating a conversation about end-of-life care and ensuring that key palliative care needs are identified. Importantly the SPICT™ requires little training and its brevity may be suited to settings in which there is limited opportunity to engage in lengthy conversations and in which clear unambiguous communication is key to timely referral and treatment. Formalising palliative care needs screening in an end-of-life conversation in acute care settings may reduce distress for patients and their informal care givers [48] and the SPICT™ is a relevant proforma for such a conversation. Furthermore with the increase in the numbers of people living with chronic illness globally [49] arguably the formal adoption of palliative care needs screening in all health care settings may not only reduce patient distress but may assist health care managers and policy makers to more appropriately plan services [50]. Identifying needs early in the illness trajectory may allow appropriate personalised care and services to be provided in a timely and cost effective manner thus avoiding health crises at the end of life [51].

Conversely caution should be exercised when recommending a tool to guide advance care planning and end of life conversations particularly in the setting of low health professional skill level. This was highlighted by experienced GP participants in the study by Afshar et al. [33]. The GPs did not consider that the SPICT-DE™ made any impact on their practice. A proforma or guideline cannot replace the need for exemplary health care professional communication during advance care planning and end of life conversations particularly as studies reporting the use of the SPICT™ were not specifically focused on testing its efficacy in this regard. Flexibility and sensitivity are required to assess and manage people with life limiting conditions to ensure care is individualised. Thus, a sufficiently trained and resourced workforce is vital in addition to aids such as the SPICT™.

In addition, although not the focus of this review we noticed that there was an apparent lack of attention paid to input from the family and consideration of the family context in the included studies. In practice the advance care planning conversation goes beyond using the family to identify palliative care needs and the requirement for referral. The conversation should include addressing family members’ concerns and emotions and facilitate communication between the person who is the focus of advance care planning and their family members [52].

There are translations of the English version of the SPICT™ available to download from the SPICT™ website for a number of countries including; Brazil, France, Greece, Portugal and South Africa. However, studies reporting the use of many versions of the SPICT™ indicates that formal validation has not been performed. Further validation may strengthen the efficacy and reputation of these versions of the tool. Further studies are required to establish the validity of translated versions of the SPICT™ in Swedish, Danish, Indonesian and the SPICT-LIS™ (Thai), for everyday use in other patient cohorts.

The SPICT™ has scope to be tested in other patient cohorts. Specifically more work is required to extend and test its use in acute care settings where the demand for palliative care is rising and appropriate timely referrals to specialist palliative care are vital to avoid unnecessary distress [51]. Similarly, there are research opportunities such as reliability and validity testing in relation to the SPICT-4ALL™ version which has been specifically designed to be used by family and informal carers.

### Strengths and limitations

This review has strengths which warrant consideration. For example, a systematic approach based on the PRISMA-SCr methodological framework was used, and the search was extensive including 5 electronic databases and many sources of grey literature. A limitation of this review is that we were unable to access a healthcare librarian to assist with the search thus important records may have been missed. In addition, we did not have funding to arrange the translation of two studies which were identified as potentially eligible. Studies included in this scoping review were not appraised for bias thus the level of evidence for the effectiveness of the SPICT™ was not reported. Of note most studies were descriptive and thus evidence for the effectiveness in relation to review question 1 (how does the tool assist with conversations about advance care planning?) is not available.

## Conclusions

The current scoping review aimed to assess the impact and extent of the use of the SPICT™. In summary the SPICT™ appears to enable advance care planning, review of care plans and initiation of palliative care in many settings. Previous research suggests that patients and their families greatly appreciate the opportunity to discuss end of life matters. The SPICT™ provides clinicians a proforma on which to base this conversation and a common language to collaborate for palliative care. Clinicians with advance care planning and end of life communication in all settings should consider using the SPICT™ for this purpose. Future research should focus on further validating the SPICT™ in more patient cohorts and acute care settings. Further testing of the tool beyond Europe in countries in Africa, Asia and North America is also warranted.

### Electronic supplementary material

Below is the link to the electronic supplementary material.

Supplementary Material 1

Supplementary Material 2

## Data Availability

Not applicable.
