# Supplementary material for: Use of the supportive and palliative care indicators tool (SPICT™) for end-of-life discussions: a scoping review
Source: BMC Palliat Care. 2024 May 16;23:119. doi: 10.1186/s12904-024-01445-z (PMC11097449; doi:10.1186/s12904-024-01445-z)
Supplement: Supplementary file 1 — Supplementary Material 1 [file 12904_2024_1445_MOESM1_ESM.docx]

**Supplementary file 2: Template of evidence extraction details**

| **Scoping Review Details** | |
| --- | --- |
| Scoping Review title: |  |
| Review objective/s: |  |
| Review question/s: |  |
| **Inclusion/Exclusion Criteria** | |
| Population: |  |
| Concept: |  |
| Context: |  |
| Types of evidence source |  |
| **Evidence source Details and Characteristics** | |
| Citation details (author, date, title, journal, volume, issue, pages) |  |
| Country |  |
| Context |  |
| Participants (age, sex, number) |  |
| **Details/Results of extracted from source of evidence (in relation to concept of ScR)** | |
|  |  |
|  |  |
| **Version of SPICT** | |

From JBI Methodology guidance for scoping reviews.
