# Supplementary material for: Use of the supportive and palliative care indicators tool (SPICT™) for end-of-life discussions: a scoping review
Source: BMC Palliat Care. 2024 May 16;23:119. doi: 10.1186/s12904-024-01445-z (PMC11097449; doi:10.1186/s12904-024-01445-z)
Supplement: Supplementary file 2 — Supplementary Material 2 [file 12904_2024_1445_MOESM2_ESM.docx]

**Supplementary file 1: Grey literature search sources and search strategy for EBSCO Medline**

International Association for Hospice and Palliative care ([www.hospicecare.com](http://www.hospicecare.com)); National Institute for Health Care Excellence (NICE) ([www.nice.org.uk](http://www.nice.org.uk)); Centre for Disease Control ([www.cdc.gov](http://www.cdc.gov)); World Health Organization ([www.who.int](http://www.who.int) ); Open Grey ([www.opengrey.eu](http://www.opengrey.eu)); Guideline Central ([www.guidelinecentral.com](http://www.guidelinecentral.com)); Grey Literature ([www.greylit.org](http://www.greylit.org)) and the Care Search Australia Grey Literature Database ([www.caresearch.com.au](http://www.caresearch.com.au)).

|  | Search strategy for EBSCO Medline database search 28.9.22 and rerun 28.1.24 |
| --- | --- |
| #1 | ((SPICT OR supportive palliative care indicators tool) AND Advance care plan AND Documentation AND Decision making AND (Conversation OR communication OR discussion) AND Patient care planning AND (Palliative care end of life care OR terminal care OR dying) AND (Chronic illness OR chronic disease)) |
|  | Find any of my search terms, find related words and find equivalent subjects included. |
| Limits | Adults 19 +  2010-September 2022 (rerun: 2010-January 2024) |
